# Follicular helper T Cells and the B cells they help

**DOI:** 10.1186/ar3936

**Published:** 2012-09-27

**Authors:** A Poholek, J-Y Choi, S Hernandez, J Weinstein, S Kim, V Bunin, J Odegard, L DiPlacido, J Craft

**Affiliations:** 1 Yale University, New Haven, CT, USA

## Background

CD4 T cells help B cells produce antibodies following antigen challenge. This response classically occurs in germinal centers (GC) located in B-cell follicles of secondary lymphoid organs (SLO), a site of immunoglobulin isotype switching and affinity maturation. GC formation requires specialized CD4 T cells, T-follicular helper (Tfh) cells, which localize to follicles and provide B cells with survival and differentiation signals that are essential for B-cell maturation into memory and long-lived plasma cells. Pathogenic autoantibodies in human and murine lupus arise in a like manner. Although Tfh cells are critical for GC development, their genesis in humans, role in promotion of autoimmunity, and potential as therapeutic targets in SLE are incompletely understood. To address these issues, we dissected Tfh cell development and function, defining their transcriptional regulation, migration, and function *in vivo *in normal and lupus-prone mice and *ex vivo *in normal humans and patients with SLE.

## Methods

We used a combination of approaches - flow cytometry, confocal microscopy, microarrays, quantitative chromatin immunoprecipitation and DNA sequencing (ChIP-seq), retroviral overexpression, and T-cell-B-cell helper assays - to characterize Tfh cells in normal mice and in lupus-prone strains, and from the tonsils of normal humans and the blood of patients with SLE.

## Results

We found that the transcription factor Bcl6 (B-cell CLL/lymphoma 6) is necessary and sufficient for Tfh development and function, via genetic control of Tfh proteins that are essential for their migration to B-cell follicles and GC and subsequent B-cell maturation. We dissected steps in Tfh development within SLO, beginning with their genesis in the T-cell zone followed by emigration to sites of B-cell interaction outside the B-cell follicle, where we have shown that B cells serve to provide signals for continued Tfh expansion and follicular migration. We have now begun to tease apart the factors that mediate T-cell-B-cell collaboration in the follicle; these represent therapeutic targets in SLE. Finally, we have shown that patients with SLE have expansion of Tfh cells in the blood, a finding that highlights their potential role in the pathogenesis of SLE and as likely therapeutic targets.

## Conclusion

These studies help define the developmental pathways for Tfh cells, and the steps that enable these cells to function in the B-cell follicle to promote immunoglobulin and autoantibody production. They have also helped define markers of Tfh cells in normals and autoimmune individuals, and suggest that they are a promising therapeutic target in patients.

